# Low Prevalence of HCV Infection Among MSM in an Intervention for HCV Micro-Elimination in Rome Urges a Focus on High-Risk Behaviours

**DOI:** 10.3390/jcm14238474

**Published:** 2025-11-28

**Authors:** Pierluca Piselli, Massimo Giuliani, Massimo Farinella, Rozenn Esvan, Alessandra Latini, Filippo Leserri, Francesco Angeli, Rosario Galipò, Elisabetta Gennaro, Alessandro Caioli, Claudia Cimaglia, Silvia Pittalis, Orfeo Bruzzi, Silvia Foracappa, Silvia Meschi, Elisa Biliotti, Elisabetta Grilli, Alessandra Nappo, Arianna Genovese, Alessia Rianda, Valentina Mazzotta, Antonio Cristaudo, Enrico Girardi

**Affiliations:** 1 National Institute for Infectious Diseases Lazzaro Spallanzani IRCCS, 00149 Rome, Italy; rozenn.esvan@inmi.it (R.E.); elisabetta.gennaro@inmi.it (E.G.); alessandro.caioli@inmi.it (A.C.); claudia.cimaglia@inmi.it (C.C.); orfeo.bruzzi@inmi.it (O.B.); silvia.meschi@inmi.it (S.M.); elisa.biliotti@inmi.it (E.B.); elisabetta.grilli@inmi.it (E.G.); alessandra.nappo@inmi.it (A.N.); alessia.rianda@inmi.it (A.R.); valentina.mazzotta@inmi.it (V.M.); enrico.girardi@inmi.it (E.G.); 2 San Gallicano Dermatological Institute IRCCS, 00144 Rome, Italy; massimo.giuliani@ifo.it (M.G.); alessandra.latini@ifo.it (A.L.); antonio.cristaudo@ifo.it (A.C.); 3 Circolo di Cultura Omosessuale “Mario Mieli” APS, 00146 Rome, Italy; m.farinella@mariomieli.org (M.F.); a.genovese@mariomieli.it (A.G.); 4 Checkpoint Plus Roma, 00182 Rome, Italy; filippo.leserri@plusroma.it; 5 Arcigay Roma, 00153 Rome, Italy; francescoangeli@hotmail.it; 6 ANLAIDS Lazio APS—Italian National Association for the Fight Against Aids, 00185 Rome, Italy; rosariogalipo@yahoo.it

**Keywords:** hepatitis C, MSM, micro-elimination

## Abstract

**Background/Objectives**: The prevalence of Hepatitis C Virus (HCV) among men who have sex with men (MSM) is not negligible; however, data from Italy—especially regarding MSM who are not infected with Human Immunodeficiency Virus (HIV)—are limited. We report data from an HCV screening programme targeted at MSM in Rome, starting in 2019 in two hospital settings and in four urban community-based (CB) settings run by non-governmental organizations (NGOs). **Methods**: Adult MSM (>18 years old) who presented for HIV or sexually transmitted infection testing, or who attended CB activities, were invited to undergo a free-of-charge rapid HCV antibody test (OraQuick HCV^®^), after providing informed consent. For all participants, demographic, clinical and behavioural data were collected using an anonymous questionnaire for all participants. Free confirmatory standard serology tests were offered for those found reactive at a rapid HCV test. Individuals with confirmed chronic HCV infection were referred through a dedicated “fast track” pathway for further clinical and laboratory assessment and direct-acting antiviral agents (DAAs) treatment according to the national treatment guidelines. **Results**: Between July 2019 and July 2023, 2714 MSM agreed to be screened for HCV infection. The median age was 36 years (interquartile range, IQR = 29–46), 91.0% were Italians, 58.0% enrolled in the two clinical centres and 10.7% reported living with HIV (people living with HIV, PLWH). Overall, 9 (0.33%) MSM tested reactive for HCV-specific antibodies using a rapid test. Eight MSM were retested and seven were confirmed to have chronic HCV infection (HCV viremia range: 8 × 10^3^–23 × 10^6^ IU/mL). The prevalence of confirmed cases was 0.26% (7/2714; 95%CI: 0.10–0.53) and was higher in PLWH compared to those not reporting HIV infection (1.04% vs. 0.17, *p* = 0.03). Four of seven confirmed HCV cases attended the STI clinic. All confirmed HCV cases reported high-risk behaviours for HCV infection and/or history of sexual transmitted infection (STI). Bening a PLWH (OR = 6.30) and current/former IDU (O = 17.02) resulted in being significantly associated with HCV infection. Other risk factors such as fisting, groupsex, chemsex and condomless anal intercourse were more common in the HCV case (OR > 2), but lacked statistical significance, likely due to small sample size. All seven individuals were linked to care, clinically assessed and started on DAAs treatment, achieving sustained viral response (SVR) in all cases. **Conclusions**: These data suggest the feasibility and potential effectiveness of a preventive programme targeting MSM living in Rome, combining HCV screening, case finding and prompt linkage to care. HCV prevalence in the screened population was lower than anticipated, although it is significantly higher in PLWH and in those with high-risk behaviours. Considering this condition of low prevalence of HCV infection among MSM in Italy, a targeted screening in PLWH and in individuals with high-risk behaviours may be more effective to achieve HCV eradication than universal screening in MSM.

## 1. Introduction

Hepatitis C Virus (HCV) infection represents a major public health problem, with an estimated 50 million people living with the infection worldwide [1]. As newly diagnosed HCV infections are typically asymptomatic, most HCV infections go undiagnosed until chronic HCV causes morbidity and severe consequences such as liver cirrhosis and hepatocellular carcinoma (HCC) [2].

The availability of direct-acting antiviral agents (DAAs) over the last ten years has transformed HCV from a chronic and serious infection to a curable disease, leading the World Health Organization (WHO) to propose a target to eliminate HCV by 2030, aiming for this date to be able to diagnose at least 90% of all infected people and to treat at least 80% of them [3]. However, the high number of deaths due to HCV—estimated at 242,000 people in 2022 [1], mostly from cirrhosis and hepatocellular carcinoma—suggests that, even if diagnosed, the number of people receiving treatment for HCV remains too low. This highlights the need to improve the linkage from diagnosis to care [3,4].

The European Association for the Study of the Liver (EASL) has proposed a micro-elimination approach to HCV eradication. In this strategy, national elimination objectives are dived into smaller, population-specific targets, where treatment and prevention efforts can be implemented more rapidly and effectively through tailored strategies [5].

Men who have sex with men (MSM) have long been recognized as a population at greater risk of HCV sexual transmission, especially among people living with Human Immunodeficiency Virus (HIV) [6,7]. Interventions targeting this group have been proposed as a part of micro-elimination strategy

Risk factors for HCV transmission in MSM are linked to unsafe sexual practices and are associated with a mix of clinical and behavioural aspects that increase the risk of infection, such as co-infection with HIV or other sexual transmitted infections (STIs), unprotected anal intercourse with multiple partners, group sex, sharing sex toys, unprotected fisting and the use of drugs to enhance sexual activity (Chemsex) [8,9,10,11,12].

In Italy, it was estimated that by January 2020 approximately 400,000 people in the general population were actively infected with HCV (overall prevalence 0.66%), of whom approximately 280,000 were undiagnosed and not connected to care [13,14].

Although HCV prevalence among HIV-uninfected MSM in Italy should not be considered negligible, information on this topic is scarce. Few interventions have been implemented to identify and treat undiagnosed HCV infection among MSM who are either HIV-negative or have an unknown HIV status [15].

In the absence of recent studies, we hypothesized that a sizeable population of MSM with undiagnosed HCV infection may exist in Italy and that these individuals may be identified through targeted screening initiatives and promptly linked to care.

Taken together, the information gathered in this project may provide a “micro-elimination” model for implementing effective interventions to pursue HCV elimination among MSM in Italy. To achieve this goal, we designed and tested an intervention to actively screen MSM for HCV infection, link them to care and treat them with DAAs, in the metropolitan area of Rome.

## 2. Materials and Methods

### 2.1. Study Design and Population

A prospective observational study, called CHIME (Conquering Hepatitis via Micro-Elimination), was conducted from 2019 to 2023 in six different settings within the metropolitan area of Rome: two clinical centres—the National Institute for Infectious Disease (INMI) and the San Gallicano Institute (ISG)—and four community-based (CB) testing sites run by non-governmental organizations (NGOs) (“Circolo di Cultura Omosessuale Mario Mieli”, “Associazione Nazionale Lotta contro l’AIDS—ANLAIDS Lazio”, Checkpoint Plus Roma and Arcigay Roma Center). To ensure the study population comprised individuals most likely to present behavioural determinants of HCV infection, screening was launched and conducted in clinical centres and community-based settings frequented by MSM who voluntarily underwent STI testing according to their self-assessed risk perception

Enrolment at the INMI included people presenting at the HIV Voluntary Counselling and Testing (VCT) site, while enrolment at the ISG included individuals seeking STI testing or clinical evaluation at the Sexual Transmitted Infections outpatient clinic.

Enrolment by NOGs occurred at community-based HIV testing sites or during special testing events held in gay venues (such as gay saunas and cruising bars) in Rome.

The CHIME study protocol was approved by the INMI Lazzaro Spallanzani Ethical Committee (approval number: 69_25, Register of Non-COVID Trials 2018). The project’s objectives were communicated through a promotional campaign called “ZeroC”, which was advertised via posters and brochures at testing locations (clinical centres and CB sites) and promoted on a dedicated website and through social media.

### 2.2. HCV Risk Assessment and Rapid Test Offer

Individuals who self-identified as MSM were offered to perform an HCV rapid test during their contact with the different setting in which the study was conducted. The inclusion criteria were as follows: male gender, age 18 years or older, reporting same-gendersexual activity in the previous 12 months and the ability to provide valid consent. The exclusion criterion was being aware of their own HCV infection or having been already tested for HCV infection within the previous 6 months. All participants received comprehensive information about the project’s objectives and signed informed consent, receiving educational materials on the risk of HCV infection and the recommended behaviours to prevent infection with STIs and HCV in particular.

Data on risk factors associated with sexual behaviours and clinical history (including previous HCV testing, HIV test results if available, presence of coinfections and previous STIs) were collected by a self-administered anonymous questionnaire. This questionnaire included factors identified by a Dutch MOSAIC study as linked to HCV acquisition, such as receptive condomless anal intercourse, group sex, sharing of sex toys and number of sexual partners in the last 6 months [16].

HCV rapid testing was performed using the OraQuick^®^ HCV Rapid Antibody Test (OraSure Technologies, Bethlehem, PA, USA), a single-use immunoassay approved by the United States Food and Drug Administration (FDA) for qualitative detection of antibodies to HCV (anti-HCV) in oral fluid, fingerstick whole blood, venepuncture or whole blood specimens. The test provides results in 20–40 min, and is characterized by a high sensitivity (ranging from 98.1% in oral fluid to 99.9% in serum) and a specificity ranging from 99.6% in oral fluid to 99.9% in fingerstick or serum and has an analytical limit of detection (LoD) of 0.75 signal-to-cutoff (s/co) for venous whole blood and 0.89 s/co for fingerstick whole blood, which means it can detect anti-HCV antibodies at these levels 95% of the time [17]. In our study, the test was performed mainly on capillary blood obtained through fingerstick using a sterile lancet, or on the oral fluid using the swab included in the test device applied to the gums (3.1% of the overall sample) following the manufacturer’s instructions.

### 2.3. Laboratory Procedures for the Confirmation Test and Molecular Characterization

In the case of the HCV reactive rapid test, HCV confirmation was performed using the ARCHITECT Anti-HCV assay, a chemiluminescent microparticle immunoassay (CMIA) designed for the qualitative detection of anti-HCV antibodies in human serum and plasma, processed on the fully automated system ARCHITECT i2000SR (Abbott Diagnostics, Wiesbaden, Germany). If the confirmation test was negative, validation was conducted using a secondary platform based on an HCV IG immunoblot, as previously described [18].

Subsequently, for all cases where HCV infection could not be excluded, HCV RNA was assessed using the Abbott RealTime HCV RNA assay (Abbott Molecular Inc., Des Plaines, IL, USA), which has a lower limit of quantitation and detection of 12 IU/mL. For samples that tested positive for HCV-RNA, the HCV genotype was determined by Abbott RealTime HCV Genotype II (Abbott Molecular, Abbott Park, IL, USA). This assay applies a dual-target real-time PCR approach, employing the 5′ UTR region to discriminate between HCV genotypes and the NS5B gene for subtyping 1a and 1b.

### 2.4. Clinical Evaluation and DAA Treatment

Individuals diagnosed with a new, untreated HCV infection were referred for pre-treatment evaluation through a dedicated fast-track assessment (FTA) to the INMI Hepatology Unit, or to the INMI HIV Unit if co-infected. When indicated, patients received a prompt initiation of DAA-treatment and were monitored during therapy according to current Italian guidelines [19].

### 2.5. Statistical Analysis

Data were described as counts and percentages for categorical variables and as median and interquartile range (IQR) for continuous variables. Significant differences between groups of interest were assessed using the Chi-square test, or Fisher’s Exact test for categorical variables, and the *t*-test or Mann–Whitney U-test for continuous variables.

Logistic regression models were used to investigate associations between relevant variables and two distinct outcomes: having been previously tested for HCV and having a confirmed reactive HCV test in the current study. Candidate predictors were selected based on clinical and epidemiological considerations, as well as established associations found in the literature. Only observations without missing data for any of the variables of interest were included in the regression model (complete case analysis). For the first outcome (being previously tested for HCV), variables significant (*p* < 0.05) in the univariable analysis were included in the multiple regression models to adjust for possible confounding effects and to assess the overall robustness of the newfound associations. The significance level was set at 0.05.

All statistical analyses were performed using Stata release 17 (StataCorp LLC, 2021; College Station, TX, USA) and R version 4.4 (R Foundation for Statistical Computing, Vienna, Austria) or SPSS version 29 (IBM Corp. Released 2023. Armonk, NY, USA: IBM Corp).

## 3. Results

A total of 2714 MSM participated in the study; of these, 1567 (57.7%) were tested in the two clinical centres and the remaining 1147 (42.3%) in the four community-based centres (see Table 1). The vast majority of participants were born in Italy (n = 2469, 91.0%), and the overall median age was 36 years (IQR: 29–46). In our sample, 291 subjects (10.7%) identified as people living with HIV (PLWH), and overall, approximately 48% of participants reported having previously been tested for HCV.

Table 2 shows the factors associated with having previously been tested for HCV. In univariate analysis, increased age (OR = 1.17 per 5-years increase), being a PLWH (OR = 6.22), having a present or former IDU sexual partner (OR = 1.89), practicing chemsex (OR = 1.60), engaging in groupsex (OR = 1.30), having any history of STI (OR = 1.89), use of pre-exposure prophylaxis (PrEP) (OR = 1.72) and being enrolled at an STI clinic site (OR = 1.66) were all associated with a higher likelihood of having been tested for HCV. In multivariate analysis, increasing age (aOR = 1.11 per 5-years increase), being a PLWH (aOR = 5.72), history of STI (aOR = 1.28), use of PrEP (aOR = 1.65) and engaging in groupsex (aOR = 1.20) were confirmed as factors independently associated with having been already tested for HCV (see Table 2).

Among all the 2714 tested individuals, 9 (0.33%) had a reactive test at enrolment. Eight out of these nine individuals returned for confirmatory testing, resulting in seven (87.5%) confirmed HCV infections, all with chronic infection and viremia ranging from 8.6 × 10^3^ to 23.7 × 10^6^ log International Unit (IU)/mL. The single person with a reactive HCV rapid test and with a negative confirmatory test was retested after six months with the same result (moderately reactive at rapid test but negative at laboratory confirmation).

Table 3 shows the characteristics of the seven identified confirmed HCV infections, resulting in an overall prevalence of chronic (confirmed) HCV infection of 0.26% (95% confidence interval, CI: 0.10–0.53%).

All HCV-positive individuals reported at least one high-risk behaviour or relevant clinical condition history. One person reported a history of intravenous drug use, four had an instance of condomless anal intercourse with their last occasional partner and six out of seven reported having been previously diagnosed with at least one STI. Within the previous six months, four had engaged in groupsex, two in chemsex and two practiced fisting (Table 3).

Three out of the seven confirmed HCV infections were identified as PLWH, with an overall prevalence among HIV-positive persons of 1.03% (95% CI: 0.21–2.98%), significantly higher than what was observed among HIV-negative persons (0.17% prevalence, 95% CI: 0.04–0.42%, *p* = 0.03). The identified genotypes were 1a (four patients) and 4 (three patients).

Following diagnosis, all individuals with confirmed chronic infection received clinical evaluation and were initiated on DAA treatment (Glecaprevir/pibrentasvir for 8 weeks, according to current guidelines) within an average of 64 days from first rapid test (range: 25–80). All treated individuals successfully achieved a sustained virologic response (SVR), defined as undetectable HCV-RNA levels 12 weeks after completing DAA therapy, according to the above-mentioned Italian guidelines.

The main factors associated with confirmed HCV infection are shown in Table 4.

As shown in Table 4, being PLWH (OR = 6.30) and being a present or former IDU (OR = 17.02) were significantly associated with HCV infection, while the proportion of individuals previously tested for HCV did not differ between the two groups. Four out of the seven confirmed HCV cases were enrolled in the STI clinic, but, despite a 5.76-times increased OR, this did not result in being significantly associated with the outcome.

Other high-risk behaviours, such as fisting, groupsex, chemsex and condomless last anal intercourse, or a history of STI, were more prevalent among subjects with HCV with OR greater than 2; however, likely due to the limited number of events, these associations did not reach statistical significance (see Table 4). The small number of confirmed cases did not allow us to perform a multivariate analysis and for this reason the analysis is underpowered to further investigate other associations. All these results could be considered cautiously.

## 4. Discussion

In this paper, we report the results of an extensive screening programme for HCV targeting MSM with unknown HCV infection in the metropolitan area of the capital of Italy, Rome. To the best of our knowledge, this is the first study conducted in Italy reporting on HCV prevalence in MSM. Out of 2714 individuals tested, we found 7 HCV-positive patients (0.26% prevalence), all of whom were linked to care and successfully treated. It should be noted that, although the prevalence of HCV in this population was lower than previously reported in other international studies [20], it aligns with the pooled HCV prevalence reported by Traeger et al. (0.38% HCV-RNA baseline prevalence among HIV-negative MSM accessing PrEP) [21]. Regarding PLWH, HCV prevalence was higher in this subpopulation compared to HIV-negative people (1.03% vs. 0.17%, *p* < 0.03).

The study population was characterized by a high rate of previous HCV testing (1299/2714, 48%), likely due to clinical conditions (e.g., PLWH), increased awareness of risks associated with sexual behaviour (e.g., group sex) or prior inclusion in screening programmes, such as those provided in the context of STI-PrEP clinics.

In our population, 5.4% (N = 147) of individuals reported having used PrEP at least once. The study began in July 2019 and ended in July 2023, before full reimbursability of PrEP in Italy (May 2023) [22], which could explain the lower percentage of PrEP users among tested individuals compared to more recent reports [23]. Interestingly, none of the seven confirmed HCV cases reported any history of PrEP use.

A substantial proportion of study participants reported behavioural risk factor such as a history of STI (54.8%), engaging in groupsex (25.1%), fisting (8.3%) and chemsex (7.0%) in the last 6 months, all factors previously found to be associated with HCV infection [10,24,25,26].

It is worth noting that, since the information concerning the above-mentioned risky behaviours was obtained via a self-administered questionnaire, responses may be influenced by the stigma that still surrounds these sexual practices, especially among PLWH [27], even though the questionnaire was anonymous.

High-risk behaviours were also reported in individuals who had a confirmed positive test for HCV (Table 3). Notably, four out of seven reported having participated in groupsex and six out of seven had a history of STIs. Despite the low prevalence of HCV infection and the small number of HCV-positive cases identified in this study which did not allow us to find significant associations with several known risk factors for HCV infection, we can still speculate, according to the literature, to support an approach of implementing HCV screening activities targeting subpopulations with specific risk factors—in this case, sexual behaviours that increase the risk of HCV infection and reinfection [28].

The HCV genotypes identified in our sample (1a and 4) are those most frequently found worldwide in MSM, which suggests shared international transmission routes of MSM-specific clusters [29]. Phylogenetic studies have shown links between local HCV infections and European clusters, highlighting the need for phylogenetic analysis of outbreaks, contact tracing, targeted testing and rapid treatment at a European level to meet WHO elimination goals [30,31]. Unfortunately sequencing of detected samples was not considered in our protocol and neither planned nor requested by the clinician according to the common guidelines; however given that the seven cases were identified throughout four years of activity and were not clustered, is unlikely that such information could have provided relevant results, apart from the identification of genotypes 1a and 4 more prevalent among MSM.

All confirmed HCV cases were chronically infected with high viremia (six out of seven, with more than 10^6^ UI/mL), despite mild or no symptoms. This finding supports the important role of these screening programmes in diagnosing highly viremic subjects who are unaware of their status, and who could otherwise contribute to the spread of infection within the MSM community.

In our study, all confirmed cases were effectively linked to treatment, resulting in definitive and permanent HCV infection resolution in 100% of subjects treated.

Our study has some limitations. First, the low observed number of confirmed HCV infections did not allow us to perform multivariate analysis, limiting the possibility to further investigate the association of specific factors and HCV infection; however, it is unlikely that performing multivariate analysis could have led us to any new or novel findings compared with larger studies among MSM.

Due to COVID-19 restrictions and/or a lower propensity to testing, especially in the initial pandemic period, we had fewer participants than expected. This required us to extend the enrollment period by an additional 18 months as well as to offer the opportunity to enrol in three other non-healthcare settings (CB Checkpoint Plus Roma, Arcigay and ANLAIDS), supported by an advertising campaign using posters, leaflets, postcards and a specific website page and social media.

Furthermore, we cannot exclude the possibility that the changes in sexual behaviour among MSM occurred during the SARS-CoV-2 pandemic when the investigation was carried out that were previously described [32,33,34] may have contributed to the lower-than-expected HCV prevalence that we observed.

Additionally, HCV testing was performed with an antibody-based test, meaning that very recent HCV infections still in the seroconversion window may have gone undetected. However, the project allowed for retesting at least six months after a previous HCV-negative test, and no seroconversion was observed in 282 out of 2714 persons enrolled (10.4%) who were retested during the campaign. A small number of enrolled individuals (3.1% overall, all enrolled in a single CB site) performed the rapid test on oral fluid, a procedure that, according to the manufacturer of the Oraquick Rapid Test, has a slightly lower sensitivity (98.1% in oral fluid vs. 99.7% in fingerstick) and comparable specificity (99.6% in oral fluid vs. 99.9% in fingerstick). However, given that none of those performing the test on oral fluid resulted preliminary positive, the impact on the final results could be considered limited.

This study identifies a subgroup of MSM engaging in high-risk behaviour (e.g., those practicing group sex, chemsex or unprotected anal intercourse, or those with a history of STI), who could benefit from a dedicated HCV screening activity. Since participants were recruited from three different settings (HIV clinics, STI clinics and CBs), a bias in the selection could have occurred that could affect prevalence estimation; the fact that four out of seven confirmed cases were enrolled by the STI clinic, however, strengthens the fact that settings which are able to identify MSM at higher risk could improve case findings. Combining DAAs with behavioural interventions specifically aimed at MSM who are HIV-negative could play a crucial role in reaching the WHO elimination targets [35]. Several studies have shown that early treatment, alongside health promotion efforts focused on reducing high-risk behaviours, is an effective strategy for controlling the HCV epidemic within the MSM population [36].

In the face of this scenario, a more structured and efficient approach would be to restrict screening to the highest-risk population, since HCV annual screening, among HIV-negative MSM, has not always been effective in identifying new HCV cases [37]. These considerations underscore the importance of maintaining a targeted approach by concentrating on subgroups within the MSM population with extremely high-risk behaviour, rather than extending HCV screening to individuals with lower-risk behaviour attending STI clinics [38].

Focusing on subgroups of individuals who consistently engage in high-risk behaviours and have a history of HCV infection—given that reinfection rates remain high among MSM—may be a more effective approach [39]. This involves developing a comprehensive strategy designed to increase HCV knowledge and awareness, encourage regular testing, ensuring rapid linkage to care, supporting risk-reduction behaviours and strengthening partner notification efforts among MSM [40].

Moreover, modelling studies indicate that targeted HCV screening among MSM, especially when integrated into HIV care or PrEP settings, can be highly cost-effective [41,42], particularly when using a “test-and-treat” approach linked to risk reduction interventions targeted at high-risk individuals [43].

These considerations are in line with those expressed by the WHO [44], which state that general HCV screening is recommended when the prevalence is 2% or more. Since, in our study, the prevalence of HCV infection is well below 2, consistent with findings from other European studies [45], HCV screening conducted systematically, especially among HIV-negative MSM, may be inefficient, while a screening based on an assessment of individual risk behaviours should rather be considered and implemented.

In conclusion, the results of this project confirm the feasibility and potential effectiveness of a micro-elimination programme targeting high-risk MSM. This approach combines HCV screening and linkage to care with prevention strategies that include behavioural interventions, elements needed to achieve the goal of HCV micro-elimination among MSM. Although the overall prevalence is very low, it remains important to continue screening MSM, regardless of HIV status, with particular attention to those who, despite engaging in high-risk behaviours, do not attend STI clinics, including those not yet on PrEP.

## Figures and Tables

**Table 1:** Baseline characteristics of the study population (n = 2714).

Characteristics	N (%)
All	2714 (100)
Age, Median (IQR)	36 (29–46)
Country of origin	
Italy	2469 (91.0)
Place of enrollment (setting)	
VCT site	923 (34.0)
STI Clinic	644 (23.7)
Community-based VCT (CBVCT)	1147 (42.3)
People living with HIV (PLWH)	291 (10.7)
Previously tested for HCV	1299 (47.9)

Abbreviations: IQR, interquartile range; VCT, voluntary counselling and testing; STI, sexually transmitted infection; HCV, Hepatitis C Virus.

**Table 2:** Factors associated with previous HCV test.

Characteristic	All (N = 2714)	Already Tested (N = 1299)	Never Tested (N = 1415)	OR (IC 95%)	aOR (IC 95%)
Age (per 5 yrs increase)—median (IQR)	36 (29–46)	39 (31–48)	34 (27–43)	**1.17 (1.13–1.21)**	**1.11 (1.07–1.15)**
Foreign born, n (%)	245 (9.0)	118 (8.1)	127 (9.0)	1.03 (0.79–1.34)	
To be PLWH, n (%)	291 (10.7)	241 (18.6)	50 (3.5)	**6.22 (4.54–8.52)**	**5.72 (4.03–8.24)**
Sexual partners in the last year—median (IQR)	8 (3–20)	5 (2–10)	4 (2–10)	1.00 (1.00–1.01)	
Present or former IDU, n (%)	27 (1.0)	18 (1.4)	9 (0.6)	2.20 (0.98–4.90)	
IDU sexual partner, n (%)	65 (2.4)	41 (3.2)	24 (1.7)	**1.89 (1.14–3.14)**	1.49 (0.87–2.58)
Fisting, n (%)	226 (8.3)	100 (7.7)	126 (8.9)	0.85 (0.65–1.12)	
Chemsex, n (%)	191 (7.0)	112 (8.6)	79 (5.6)	**1.60 (1.18–2.15)**	1.27 (0.91–1.77)
Groupsex, n (%)	680 (25.1)	359 (27.6)	321 (22.7)	**1.30 (1.09–1.55)**	**1.20 (1.00–1.45)**
Unprotected last anal intercourse with occasional partner, n (%)	725 (26.7)	329 (25.3)	396 (28.0)	0.87 (0.74–1.04)	
Previous STI, n (%)	1488 (54.8)	818 (63.0)	670 (47.3)	**1.89 (1.62–2.20)**	**1.28 (1.08–1.52)**
Previous PrEP, n (%)	147 (5.4)	89 (6.8)	58 (4.1)	**1.72 (1.23–2.42)**	**1.65 (1.16–2.35)**
Use of recreational drugs, n (%)	910 (33.5)	454 (34.9)	456 (32.2)	1.03 (0.88–1.21)	
Setting (VCT site)	923 (34.0)	406 (31.3)	517 (36.5)	Ref	Ref
STI Clinic	644 (23.7)	364 (28.0)	280 (19.8)	**1.66 (1.35–2.03)**	0.81 (0.63–1.03)
Community-based VCT (CBVCT)	1147 (42.3)	529 (40.7)	618 (43.7)	1.09 (0.92–1.30)	1.09 (0.92–1.31)

Abbreviations: HCV, Hepatitis C Virus; OR, odds ratio; aOR, adjusted OR; IQR, interquartile range; PLWH, people living with HIV; IDU, injecting drugs users; STI, sexually transmitted infection; PrEP, pre-exposure prophylaxis; VCT, voluntary counselling and testing. OR or aOR with *p*-value < 0.05 are highlighted in bold.

**Table 3:** Overview of patients with positive HCV antibody results during screening.

Patient	HIV	High-Risk Behaviours	Viremia (IU/mL)	Genotype	Days from HCV-Testing to DAA Treatment	Outcome
1	Neg	GS-F-pSTI	5.4 × 10^6^	1a	35	SVR
2	Pos	GS-pSTI	1.7 × 10^6^	4	88	SVR
3	Pos	GS-F-UAI-pSTI	3.1 × 10^6^	1a	47	SVR
4	Pos	UAI	23.7 × 10^6^	1a	67	SVR
5	Neg	UAI-pSTI	4.3 × 10^6^	1a	42	SVR
6	Neg	pSTI	8.6 × 10^3^	4	25	SVR
7	Neg	GS-IDU-Chem-UAI-pSTI	8.7 × 10^6^	4	80	SVR

Abbreviations: HCV, Hepatitis C Virus; HIV, Human Immunodeficiency Virus; IU, International Unit; DAA, direct action antiretrovirals; GS: groupsex; F: fisting; UAI: unprotected last anal intercourse with an occasional partner; IDU: injecting drug user; Chem: chemsex; pSTI: previous sexual transmitted infection; SVR: sustained virologic response.

**Table 4:** Factors associated with positive HCV result.

Characteristics	All (N = 2713) ^a^	HCV-Pos (N = 7)	HCV-Neg (N = 2706)	OR (IC 95%)
Age (per 5 years increase)—median (IQR)	36 (29–46)	46 (42–48)	36 (29–46)	1.29 (0.96, 1.72)
To be PLWH, n (%)	291 (10.7)	3 (42.9)	288 (10.7)	**6.30 (1.40–28.30)**
Foreign born, n (%)	2468 (91.0)	5 (71.4)	2463 (91.0)	4.09 (0.79–21.21)
Sexual partners in the last year—median (IQR)	8 (3–20)	40 (10–55)	8 (3–20)	1.01 (0.99–1.02)
Present or former IDU, n (%)	27 (1.0)	1 (14.3)	26 (1.0)	**17.02 (2.00–147.80)**
IDU sexual partner, n (%)	65 (2.4)	1 (14.3)	64 (2.4)	6.88 (0.82–58.00)
Fisting, n (%)	225 (8.3)	2 (28.6)	223 (8.2)	4.43 (0.86–22.98)
Chemsex, n (%)	191 (7.0)	1 (14.3)	190 (7.0)	2.21 (0.26–18.43)
Groupsex, n (%)	680 (25.1)	4 (57.1)	676 (25.0)	4.01 (0.89, 17.94)
Unprotected last anal intercourse with occasional partner	725 (26.7)	4 (57.1)	721 (26.6)	3.67 (0.82–16.45)
Previous STI, n (%)	1488 (54.9)	6 (85.7)	1482 (54.8)	4.96 (0.60–41.23)
Previous HCV-negative test, n (%)	1299 (47.9)	3 (42.9)	1296 (47.9)	0.82 (0.18–3.65)
Previous PrEP, n (%)	147 (5.4)	0 (-)	147 (5.4)	NC
Use of recreational drugs, n (%)	909 (33.5)	4 (57.1)	905 (33.4)	2.29 (0.51–10.27)
Setting (VCT site)	923 (34.0)	1 (14.3)	922 (34.1)	Ref
STI Clinic	644 (23.7)	4 (57.1)	640 (23.6)	5.76 (0.64–51.67)
Community-based VCT (CBVCT)	1146 (42.2)	2 (28.6)	1144 (42.3)	1.61 (0.15–17.79)

^a^ The analysis was conducted on 2713 persons, after exclusion of the participant with a non-confirmed HCV test. Abbreviations: HCV, Hepatitis C Virus; OR, odds ratio; IQR, interquartile range; PLWH, people living with HIV; IDU, injecting drugs users; STI, sexually transmitted infection; PrEP, pPre-eExposure pProphylaxis; VCT, voluntary counselling and testing; NC, Not computed. OR with *p*-value < 0.05 are highlighted in bold.

## Data Availability

Data are available upon reasonable request from the corresponding author.
